# Economic Burden Associated with Head Louse (*Pediculus humanus capitis*) Infestation in Iran

**DOI:** 10.18502/ijph.v49i7.3589

**Published:** 2020-07

**Authors:** Mojtaba SALIMI, Abedin SAGHAFIPOUR, Hadi HAMIDI PARSA, Majid KHOSRAVI

**Affiliations:** 1. Research Center for Environmental Determinants of Health, Kermanshah University of Medical Sciences, Kermanshah, Iran; 2. Department of Public Health, Faculty of Health, Qom University of Medical Sciences, Qom, Iran; 3. Deputy of Management and Resources Development, Qom University of Medical Sciences, Qom, Iran

**Keywords:** Head louse, *Pediculus humanus capitis*, Infestation, Iran

## Abstract

**Background:**

The head louse infestation is a public health issue in the world especially, affecting most people who live in camps, school-aged children and their families. Head lice treatment has economic ramifications that often under calculated. The aim of this study was evaluation of economic burden associated with head louse infestation in Iran.

**Methods:**

In a cross-sectional study, 500,002 infestations were diagnosed among suspected head lice infested people who referred to health care system in all provinces of Iran during 2017. Direct and indirect costs related to paid by patients and government systems were extracted by referring to accounting documents and interviews with patients and experts and were recorded in researcher-made forms. Microsoft Excel 2010 software was used for economic burden calculation.

**Results:**

The incidence rate of head lice infestation in Iran was 500,002/79,926,270 (625.5 per 100,000 populations). Economic burden of head lice in the country was calculated at 5,790,143$. Direct and indirect costs, governmental cost, out of pocket and total costs of head lice were included 3.14$, 2.84$, 5.98$, 5.60$ and 11.58$ per case respectively.

**Conclusion:**

The direct and indirect costs associated with treatment of infestations were relatively high. Therefore, the creation of medical facilities such as availability of diagnostic and treatment strategies can be effective in the control of infestation. The adoption of infestation prevention methods, such as health education to people at risk of infestation, reduces the incidence of head lice and imposition of related treatment costs on governmental health care system and head lice cases.

## Introduction

The head louse, *Pediculus humanus capitis* (Phthiraptera: Pediculidae) is an obligate ectoparasite of placental mammals especially humankind. This blood-feeding insect does not survive away from the body’s host or loses its infestation (1). Human head lice infestation (pediculosis) is a parasitic health problem, which is prevalent throughout the world, even in developed countries (2). The prevalence of this public health issue is not limited to specific socioeconomic conditions and is found at all socioeconomic groups of society, but it more prevalent in places where human population density is higher with less access to primary health services such as schools, camps and so on (3). Head louse usually is transmitted from one person to another via head to head contact directly and via in animatic objects (4). Although this infestation does not transmit any disease, it can decrease the quality of life. In addition, the insect as an ectoparasite is often injected salivary proteins into the patient’s skin because of several types of blood feeding during the day. It leads to problems such as sensitivity, fatigue, insomnia, skin lesions and discomfort. Occasionally, acute allergic reactions, such as intense itching, are caused followed by repeated injections of lice saliva (5).

Currently, head lice infestation has worldwide distribution and has been occurred in many areas of the world such as the United States (6–12 million per year), Argentina, Brazil, the United Kingdom, Australia and Turkey (6). Despite the substantial costs of care associated with head lice infestation in Iran, the prevalence of pediculosis in children and adolescents in Iran is still high (7). Many studies have done about the prevalence of head lice infestations in different areas and cities of Iran (8–10), but a valid data on how many populations get head lice each year in Iran are not reported; however, according to anecdotal reports of Iranian Centers for disease Control and Prevention (CDC), 500,000 infestations happen in Iran annually. A recent study evaluated 581 per 100,000 infestations occur in Iran among children and adolescents (7).

The calculation of the economic cost associated with disease is used to determine the burden of disease in societies. Totally, economic costs of care associated with head lice infestation are included direct and indirect costs. Direct costs due to diagnosis and treatment of disease and indirect costs related to lost time from work and school (11). In addition, lost wages for parents, costs of daycare for parents who have to employ nurses at home because they cannot miss work are included as indirect costs. The expense of misdiagnosis, treatment failure, and overuse of pediculicide agents must also be added as contributors to economic burden of treating head lice infestation not calculated. Evaluation of these contributors is more difficult to quantify. The costs of diagnosing and treating of diseases can have a huge impact on the household economy and the health care system. Therefore, the creation of facilities such as the availability of diagnostic and treatment methods along with getting accurate information on the economic burden of this health problem can help health decision-makers to estimate the size of this health issue and the economic benefits of preventing the disease (12, 13). As a result, allocate sufficient funds to prevent and control of infestation. In recent years, head lice epidemic has become a major health issues and social economic problem that has led to the involvement of the healthcare systems and societal problem with substantial costs in different areas of Iran (8, 9).

This study aimed to estimate the economic burden of head lice in all provinces of Iran.

## Materials and Methods

### Study area

This study was carried out in all 31 provinces of Iran. Iran is located in the southwest of Asia and in the Middle East. The country, located between 25° 03′−39° 47′ E and 44° 05′−63° 18′ N with an area of 1, 648, 195 km^2^. Based on the most recent census in 2016, the country has a population of approximately 79,926,270 out of which 68.4% inhabit in urban areas and 31.6% in rural environs (14, 15). Head lice infestation is an endemic health issue in all areas of the country (3). Ethical clearance was earned from the Institutional Ethics Committee of Qom University of Medical Sciences (QUMS.REC.1396.118).

### Data collection

In this cross-sectional study, 500,002 infestations were detected among suspected head lice infested people who referred to health care system in all provinces of Iran during 2017. The patients’ diagnosis and treatment data of head lice infested cases from provincial health centers were reported to CDC of the Iranian Ministry of Health. All infested people were treated with 1% permethrin shampoo; two applications almost 7 to 10 d apart are generally recommended for treatment and were examined after the end of the treatment period. Eradication of all lives lice and remove actual eggs (nits) after two applications almost a week apart was considered as treatment success (16). The costs of health care services to infested head lice cases were taken of the finance department of the Center for Disease Control and Prevention (CDC), Ministry of Health and Medical Education (Iran).

### Data analysis

After holding several meetings with the technical deputy of the CDC, experts in the prevention & control diseases, network expansion plan and pharmacy departments, data has been obtained and recorded in the researcher-made forms. Then the financial records were returned to the finance department of CDC. The collected data were entered into Microsoft Excel 2010 software. Ultimately, direct and indirect costs associated with health care to patients were calculated. Direct costs due to diagnosis and treatment of infected people were included expenses related to out of pocket (doctor visits for prescription lice treatments, medical materials and supplies), personal costs, providing head lice treatment products such as permethrin shampoo as a first-line choice for treating head lice infestation. Indirect costs related to lost time from work and school. In addition, lost wages for parents, costs of day-care for parents who have to employ nurses at home because they cannot miss work are included as indirect costs. Expenses related to consumable materials and supplies (note books and stationery), public utility costs (water, electricity, telephone) should be added to indirect costs. In addition, overhead costs include building maintenance costs (cooler, building and equipment repairs, etc.), transportation costs and depreciation costs of buildings and equipment. Microsoft Excel 2010 software was used for the costs calculation.

## Results

Totally, 500,002/79,926,270 (625.5 per 100,000 populations) infested head lice patients were diagnosed and recorded among suspected cases to human head lice infestation who referred to health care system in all province of Iran during 2017.

**Fig. 1:**
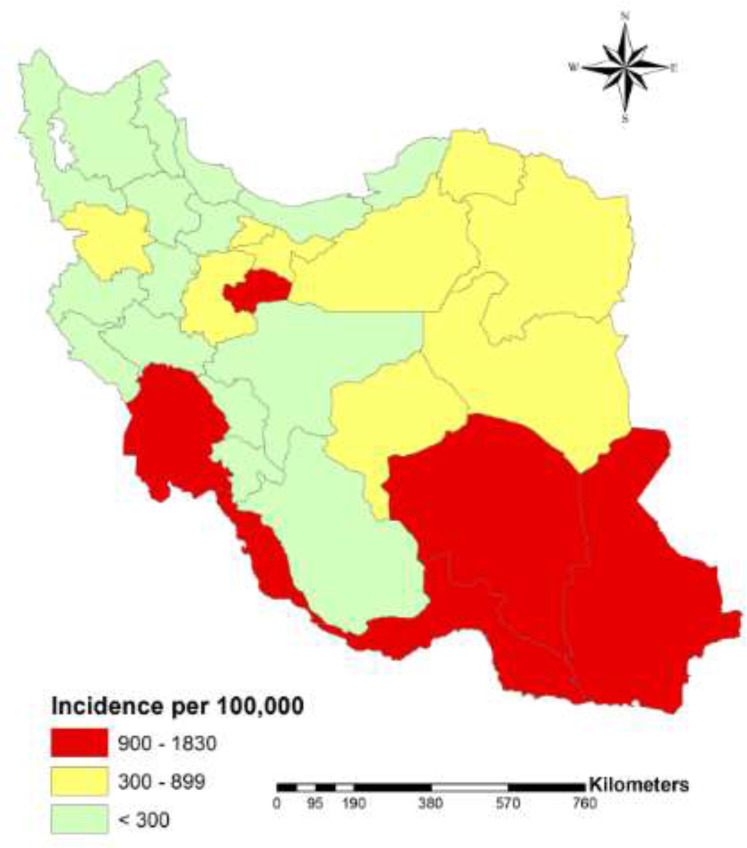
Spatial distribution of head lice infestation in Iranian people during 2017

The infestations were higher in six provinces including Kerman, Hormozgan, Sistan & Baluchestan, Khuzestan, Bushehr, and Qom, mainly located in southeast, south and central Iran (Table 1, Fig. 1).

**Table 1:** Some provinces with high Incidence of head lice infestation among Iranian people in 2017

*** Provinces ***	*** Population (number) ***	*** Frequency of cases (number) ***	*** Incidence per 100,000 ***
Kerman	3,165,000	57,919	1830
Hormozgan	1,776,415	25,331	1426
Sistan & Baluchestan	2,775, 014	38,877	1401
Khuzestan	4,710,509	61,707	1310
Bushehr	1,163,400	11,424	982
Qom	1292000	12,594	975
Tehran	13,267,637	119,276	899
Markazi	1,429,475	12,193	853
South Khorasan	768,898	5,605	729
Razavi Khorasan	6,434,501	38,671	601
North Khorasan	863,092	4,867	564
Yazd	1,138,533	4,781	420
Semnan	702,360	2,212	315
Kurdistan	1,603,011	4,953	309
Alborz	2,712,400	8,137	300
All of remain provinces	36,122,025	91,455	253
Total (Iran)	79, 926, 270	500002	625.5

The economic burden of all infestations was estimated at 289,507,158,024 rials (5,790,143$) over a one-year period in the country. On average, direct costs of providing health care services to all head lice infested cases were calculated for each person (Expenditure per capita) was 157,337 (3.14$).

Indirect costs (overhead) per person were about 141,675 rials (2.84$), the governmental cost (direct & indirect costs) per patient was 299,012 rials (5.98$), out of pocket was about 280, 000 rials (5.6$) for each patient, and the average total cost during this period was estimated at 579,012 (11.58$) rials per case.

Direct and indirect costs related to this public health problem were close to each other, so that 39,630$ (52.6%) of the costs were direct costs and 35,685$ (47.4%) also included the cost of overhead treatment for head lice infestation (Table 2). The ratio of direct to indirect costs was 1.11. Other results of calculation of the cost of care related to head lice infestation in this area were presented in Tables 2, 3.

**Table 2:** Estimation of direct, indirect, governmental, out of pocket and total costs of health care to head lice infested people in Iran, 2017

*** Health care costs *** *** Major costs groups ***	*** Total costs of cases (500002/79,926270) rials ($) ***	*** Expenditure per capita rials ($) ***
Direct costs	78,668,814,674 (1,573,376)	157,337 (3.14)
Indirect costs (overhead)	70,837,783,350 (,1416,755)	141,675 (2.84)
Governmental cost	149,506,598,024 (75,315)	299,012 (5.98)
Out of pocket	140,000,560,000 (70,526)	280,000 (5.60)
Total costs (Governmental+ Out of pocket)	289,507,158,024 (5,790,143)	579,012 (11.58)

**Table 3:** Elements of governmental costs associated with head lice infested people in Iran, 2017

*** Major cost groups ***	*** Total cost rials ($) ***	*** Expenditure per capita rials ($) ***
Paying salaries to health-care providers	14,803,059,212 (296,061)	29,606 (0.59)
Medical materials and devices consumed	63,865,755,462 (1,277,315)	127,731 (2.55)
Building depreciation and physical spaces costs	22,560,590,242 (451,211)	45,121(0.90)
Administrative, health and treatment facilities costs	9,091,536,366 (181,830)	18,183(0.37)
Urban amenities costs	39,185,656,742 (783,713)	78,371(1.57)
Total	3,765,752,198 (75,315)	299,012 (5.98)

## Discussion

In present study, prevalence of head lice infestation calculated at 625.5 per 100,000 populations (0.62%), which was relatively lower than the incidence rate in the Asian countries (varied from 0.7% to 59%) (6). Head lice infestations were higher in six provinces that mainly were located in southeast, south and central Iran. Pediculosis is prevalent in most areas of the world (17). In Iran, the incidence of this health problem is relatively high, the incidence of head lice was estimated in the population of children and adults in Shiraz and Kerman cities (southern Iran) 1/100,000 to 8,303/100,000 respectively and in the total Iranian population was 581 cases per 100,000 people (7). Head lice infestation has relation with various ecological factors (individual, social, economic, cultural, climatic, etc.) (18). Climatic and social factors such as hot and dry climates and high population density, especially in schools, to provide a suitable breeding place for head lice growth and its transmission as head to head contact directly. Estimating the economic burden of a disease to be done to quantify the importance of disease in human populations. Furthermore, these types of analyzes help politicians and decision-makers at a macro level to determine the priority of allocating funds for disease prevention and control (19). In Iran, many studies have been done to estimate the economic burden of diseases and health problems (20, 21). For instance, costs for cases of scorpionism and snakebite were 50,656,424 $ and 11,317,416 $, respectively (20). Nevertheless, in the case of calculating the economic burden of head lice infection, no studies have been conducted in Iran. In this present study, economic burden of head lice was calculated at 289,507,158,024 rials (5,790,143$). Direct and indirect costs, governmental cost, out of pocket and total costs (governmental+ out of pocket) of head lice were included 157,337 rials (3.14$), 141,675 rials (2.84$), 299,012 rials (5.98$), 280,000 rials (5.60$) and 579,012 rials (11.58$) per case respectively. In the world, there are few studies on the estimated costs of health services provided to people with head lice infestation, all of which have a significant financial burden on patients and their health care systems (11, 22, 23). Some of these health services are included doctor visits for diagnosis and guidance, lice products, prescription lice treatments, hair-brushes, combs and hair accessories (11). Head lice treatment costs and demonstrated economic burden of this health problem was analyzed. Direct and indirect costs of treatment such as costs of pediculicides (Lindane, Malathion 0.5%, so on), diagnostic and treatment practices and lost school days were mentioned as economic burden of head lice in the United States (11,22). In addition to cost of the pharmacoeconomic burden of head lice treatment, there are many considerable costs include loss of income, cost of doctor visits, out of patients pocket to transport to the physician office and so on must be considered in recovery process related to head lice infested people (23).

One of the limitations of this study was the failure to calculate cost of patients’ lost time. Lost school days and lost their work days that head lice infested people must stay home to rest and care for children came back home from schools because of their infestations should be added. In addition, other intangible costs were costs of mental disorders due to this health problem were not considered in the study that is another limitation of the study.

## Conclusion

Direct and indirect costs associated with the diagnosis and treatments of head lice infestation are high in Iran. Therefore, considering prevention and control strategies such as health education to people at risk of this health issue about types of human head lice transmission and early detection of this infestation, can reduce the costs on patients and health care system. In general, this plan is more affordable. In the head lice infestation program, facilities such as the free-of-charge diagnosis and health planners and policy makers will target treatment strategies to reduce out of pocket.

## Ethical considerations

Ethical issues (Including plagiarism, informed consent, misconduct, data fabrication and/or falsification, double publication and/or submission, redundancy, etc.) have been completely observed by the authors.

## References

[1] Bonilla DL Durden LA Eremeeva ME Dasch GA (2013). The Biology and Taxonomy of Head and Body Lice-Implications for Louse-Borne Disease Prevention. PLoS Pathog, 9(11): e1003724. 24244157 10.1371/journal.ppat.1003724 PMC3828170

[2] Sangaré AK Doumbo OK Raoult D (2016). Management and Treatment of Human Lice. Biomed Res Int, 2016:8962685. 27529073 10.1155/2016/8962685 PMC4978820

[3] Moosazadeh M Afshari M Keianian H Nezammahalleh A Enayati AA (2015). Prevalence of Head Lice Infestation and Its Associated Factors among Primary School Students in Iran: A Systematic Review and Meta-analysis. Osong Public Health Res Perspect, 6(6):346–56. 26835244 10.1016/j.phrp.2015.10.011 PMC4700766

[4] Burkhart CN Burkhart CG (2007). Fomite transmission in head lice. J Am Acad Dermatol, 56: 1044–7. 17187895 10.1016/j.jaad.2006.10.979

[5] Canadian Paediatric Society (2004). Head lice infestations: A clinical update. Paediatr Child Health, 9(9):647–651. 19675856 10.1093/pch/9.9.647 PMC2724133

[6] Falagas ME Matthaiou DK Rafailidis PI Panos G Pappas G (2008). Worldwide Prevalence of Head Lice. Emerg Infect Dis, 14(9):1493–1494. 18760032 10.3201/eid1409.080368 PMC2603110

[7] Amirkhani MA Alavian SM Maesoumi H (2011). A Nationwide Survey of Prevalence of Pediculosis in Children and Adolescents in Iran. Iran Red Crescent Med J, 13(3):167–170. 22737457 PMC3371940

[8] Saghafipour A Nejati J Zahraei-Ramazani A (2017). Prevalence and Risk Factors Associated with Head Louse (Pediculus humanus capitis) in Central Iran. Int J Pediatr, 5(7): 5245–5254.

[9] Firoozfar F Moosa-Kazemi S H Bahrami A (2019). Head Lice Infestation (Pediculus humanus capitis) Prevalence and Its Associated Factors, Among The Kormanj Tribes in North Khorasan Province. Shiraz E Medical Journal, 20(4):e80292.

[10] Nejati J Keyhani A Tavakoli Kareshk A (2018). Prevalence and Risk Factors of Pediculosis in Primary School Children in South West of Iran. Iran J Public Health, 47(12):1923–1929. 30788308 PMC6379608

[11] Hansen RC O’Haver J (2004). Economic Considerations Associated with Pediculus humanus capitis Infestation. Clin Pediatr (Phila), 43 (6):523–7. 15248004 10.1177/000992280404300603

[12] Adhikari SR Maskay NM (2003). The economic burden of Kala-azar in households of the Danusha and Mahottari districts of Nepal. Acta Trop, 88(1):1–2. 12943969 10.1016/s0001-706x(03)00156-6

[13] Uranw S Meheus F Baltussen R Rijal S Boelaert M (2013). The Household Costs of Visceral Leishmaniasis Care in South-eastern Nepal. PLoS Negl Trop Dis, 7(2): e2062. 23469298 10.1371/journal.pntd.0002062 PMC3585119

[14] Abedi-Astaneh F Hajjaran H Yaghoobi-Ershadi MR (2016). Risk Mapping and Situational Analysis of Cutaneous Leishmaniasis in an Endemic Area of Central Iran: A GIS-Based Survey. PLoS One, 11(8): e0161317. 27574805 10.1371/journal.pone.0161317 PMC5004885

[15] Akbarzadeh K Saghafipour A Jesri N (2018). Spatial Distribution of Necrophagous Flies of Infraorder Muscomorpha in Iran Using Geographical Information System. J Med Entomol, 55(5):1071–1085. 29982597 10.1093/jme/tjy098

[16] Amirkhani MA Aminaei T Ardalan G Dashti M Islami M Jamali M (2009). Guideline to prevention and treatment of lice infestation. 1st ed Iran: Seda Publishing Center p. 23–24.

[17] Cummings C Finlay JC MacDonald NE (2018). Head lice infestations: A clinical update. Paediatr Child Health, 23(1):e18–e24. 29479286 10.1093/pch/pxx165 PMC5814977

[18] Willems S Lapeere H Haedens N (2005). The importance of socio-economic status and individual characteristics on the prevalence of head lice in schoolchildren. Eur J Dermatol, 15(5):387–92. 16172050

[19] Devleesschauwer B Maertens de Noordhout C Smit GSA (2014). Quantifying burden of disease to support public health policy in Belgium: opportunities and constraints. BMC Public Health, 14:1196. 25416547 10.1186/1471-2458-14-1196 PMC4246467

[20] Mashhadi I Kavousi Z Peymani P (2017). Economic Burden of Scorpion Sting and Snake Bite from a Social Perspective in Iran. Shiraz E-Medical Journal, 18(8): e57573.

[21] Heydarpour F Akbari Sari A Mohebali M Bokaie S (2017). Economic Burden of Cutaneous and Visceral Lishmaniasis in Iran in 2013. irje, 13 (1) :1–13.

[22] West DP (2004). Head lice treatment costs and the impact on managed care. Am J Manag Care, 10(9):S277–82. 15515633

[23] Bubik RJ Cices A Huynh T (2017). The unique pharmacoeconomic burden in managing head lice infestation. J Am Acad Dermatol, 76(6): AB257, JUN.

